# Genetic Polymorphisms and Endocrine Pathways Associated with Problematic Digital-Media Use and Gaming Disorder: A Narrative Review

**DOI:** 10.3390/jpm16100500

**Published:** 2026-09-27

**Authors:** Evangelia Boumpalou, Alexia Papageorgiou, Georgios Damianos, Theodoros N. Sergentanis, Artemis K. Tsitsika

**Affiliations:** 1 Postgraduate Programme “Strategies of Developmental and Adolescent Health”, Medical School, National and Kapodistrian University of Athens, 11527 Athens, Greece; evelinamp@uoa.gr; 2 Second Department of Pediatrics, “Panagiotis & Aglaia Kyriakou” Children’s Hospital, National and Kapodistrian University of Athens, 11527 Athens, Greece; papageorgiou.2161680@studenti.uniroma1.it (A.P.); gdamianos@department.uoa.gr (G.D.); 3 Department of Public and Community Health, University of West Attica, 11521 Athens, Greece; tsergentanis@uniwa.gr

**Keywords:** problematic digital-media use, gaming disorder, dopamine, genetic polymorphisms, HPA axis, cortisol, *DRD2*, *COMT*, adolescents

## Abstract

**Background**: Problematic digital-media use and gaming disorder have emerged as important public health concerns, particularly among adolescents. Along with psychosocial factors, genetic susceptibility and neuroendocrine pathways may contribute to individual vulnerability to problematic digital-media use and gaming-related problems. **Objectives**: This review aims to summarize current evidence on the genetic polymorphisms and endocrine pathways associated with problematic digital-media use and gaming disorder, with emphasis on dopaminergic reward mechanisms and hypothalamic–pituitary–adrenal (HPA) axis regulation. **Methods**: A structured literature search was conducted in PubMed/MEDLINE, Scopus, and Google Scholar for studies published up to 30 June 2026. Relevant human studies addressing genetic, epigenetic, endocrine, or related neurobiological mechanisms of problematic digital-media use and gaming-related behaviors were identified and narratively synthesized according to the principal biological pathways examined. **Results**: The available evidence suggests that genetic variants affecting dopaminergic neurotransmission (*DRD2*, *COMT*, *DAT1*, and *DRD4*), stress regulation (*CRHR1*, *FKBP5*), serotonergic signaling (*SLC6A4*), sleep-circadian regulation and other pathways (*CHRNA4*, *NTRK3*) may increase susceptibility to problematic Internet and gaming use. Excessive digital media exposure has been associated with alterations in dopaminergic signaling, stress and sleep–circadian regulation, including changes in hypothalamic–pituitary–adrenal axis activity, cortisol secretion and melatonin secretion. These alterations may be related to differences in reward processing, sleep, and emotional regulation, as well as to compulsive digital behaviors. Bidirectional associations have also been reported between problematic digital-media use and depression, anxiety disorders, and attention-deficit/hyperactivity disorder (ADHD). **Conclusions**: Genetic and endocrine factors may contribute to a broader biopsychosocial vulnerability to problematic digital-media use and gaming-related problems. However, current evidence is insufficient to establish individual genetic variants or endocrine measures as clinically actionable biomarkers. Future research should prioritize large, adequately powered longitudinal studies with harmonized phenotypes, objective behavioral measures, genome-wide approaches, and independent replication to clarify the clinical relevance of biological markers and their potential role in risk prediction and personalized intervention.

## 1. Introduction

Digital technology use encompasses a broad range of behaviors, including internet use, online gaming, smartphone use, social-media engagement, and other forms of screen-based activity. These behaviors should not be considered interchangeable, as they differ in their clinical characteristics, diagnostic status, underlying mechanisms, and available evidence. Accordingly, the present review distinguishes gaming disorder from other forms of problematic digital-media use and considers the available evidence for each behavioral domain separately.

Internet addiction has been used to describe excessive and poorly controlled engagement with internet-based activities that may result in psychological distress or impairment in everyday functioning. The related concept of Problematic Internet Use (PIU) generally refers to problematic and compulsive use of digital media, characterized by loss of control over time spent online, neglect of social and professional obligations, and the occurrence of withdrawal symptoms (such as anxiety and irritability) when disconnected. Depending on the individual and the context, PIU may involve compulsive scrolling of social media, excessive gaming and online browsing, despite harmful side effects [1].

Smartphone addiction often manifests through repetitive checking, persistent social media use, fear of missing out (FOMO), and interference with sleep or daily activities. Although it is not currently recognized as a distinct formal clinical disorder in the DSM-5 or ICD-11, it has been investigated in relation to behavioral addiction-related constructs, including impaired self-regulation, compulsive use, and altered reward-related processes [2].

Gaming disorder (GD) is characterized by impaired control over gaming, a progressive prioritization of gaming over other interests and daily activities, and persistent gaming despite adverse consequences. For a diagnosis to be established, the behavior pattern must be sufficiently severe to cause significant impairment in personal, family, social, educational, occupational, or other important areas of functioning. The relevant pattern of gaming behavior and associated features is generally present for at least 12 months, although a shorter duration may be considered when all diagnostic requirements are met and the symptoms are severe. The World Health Organization formally recognized gaming disorder in the International Classification of Diseases (ICD-11), emphasizing its clinical significance. In contrast, the Diagnostic and Statistical Manual of Mental Disorders (DSM-5) introduced Internet Gaming Disorder (IGD) as a condition for further study. According to DSM-5, IGD is characterized by nine symptoms: (1) preoccupation with gaming, (2) withdrawal, (3) increased tolerance to gaming, (4) unsuccessful attempts to control or reduce gaming, (5) loss of interest in other hobbies or activities, (6) continued excessive use, (7) deception regarding the extent of gaming, (8) use of gaming to escape or relieve negative mood, and (9) jeopardizing career or relationships [3]. In the DSM-5-TR, gambling disorder is currently the only behavioral addiction formally classified within the category of substance-related and addictive disorders, whereas IGD is included as a condition requiring further research [4].

Dopaminergic signaling has been implicated in problematic digital-media use through its roles in reward prediction, motivational salience, cue-triggered wanting and reinforcement learning. These processes may contribute to repeated and habitual engagement with digital platforms and games, particularly in vulnerable individuals [5,6].

Genetic variation may represent one source of such individual differences. It encompasses a broad range of differences in DNA sequence, including single-nucleotide variants, sequence repeats, insertions and deletions, and larger structural changes. Single-nucleotide polymorphisms (SNPs) constitute one of the most extensively studied forms of genetic variation and may occur in either coding or non-coding genomic regions. Depending on their location and functional context, genetic variants may have minimal biological effects or may influence gene expression, RNA processing, protein structure, receptor function, or other molecular processes. Molecular genetic research in Internet use disorders and related behavioral phenotypes has therefore examined whether variation in genes involved in neurotransmitter and neuropeptide systems may contribute to differences in susceptibility to problematic digital behaviors [7,8,9].

Although psychological, social, and environmental factors play important roles in the development and persistence of problematic digital-media use and gaming disorder [10], increasing evidence indicates that biological factors may also contribute to individual differences in vulnerability. Genetic variation and endocrine mechanisms may influence these behaviors through pathways involving neurotransmitter signaling, hormonal regulation, stress responses, reward processing, and sleep–circadian regulation. A better understanding of these biological pathways may contribute to the development of more informed approaches to prevention, early identification, and clinical management.

Accordingly, this narrative review examines the potential associations between genetic polymorphisms, endocrine responses and problematic digital-media use and gaming disorder. Particular attention is given to dopaminergic pathways, stress-related endocrine mechanisms, and sleep–circadian regulation, while recognizing the distinct clinical and diagnostic characteristics of the behavioral domains considered.

## 2. Methods

A structured literature search was conducted in PubMed/MEDLINE, Scopus and Google Scholar to identify studies examining genetic polymorphisms and endocrine or neurobiological pathways potentially associated with problematic digital-media use and gaming-related behaviors. The search covered the available literature published up to 30 June 2026.

The search strategy combined terms related to problematic digital-media use and gaming with terms referring to genetic variation and relevant biological pathways. The search terms included combinations of the following concepts: (“problematic internet use” OR “internet addiction” OR “problematic digital-media use” OR “problematic smartphone use” OR “social media addiction” OR “internet gaming disorder” OR “gaming disorder” OR “problematic online gaming”) AND (“genetic polymorphism” OR polymorphism OR genotype OR allele OR “genetic association” OR epigenetic* OR methylation) AND (dopamine OR DRD2 OR DRD4 OR COMT OR DAT1 OR SLC6A3 OR serotonin OR SLC6A4 OR stress OR HPA OR CRHR1 OR FKBP5 OR melatonin OR circadian OR endocrine).

Studies were considered eligible when they (i) investigated problematic digital-media use, Internet-related addictive behaviors, Internet gaming disorder, gaming disorder or closely related problematic digital behaviors, (ii) examined a genetic, epigenetic, endocrine, neurobiological or biologically relevant pathway, (iii) reported original human data and (iv) were published in English. Studies involving adolescents, young adults or mixed-age populations were considered, provided that the investigated phenotype was relevant to the scope of the present review.

Studies were excluded when they (i) did not investigate a digital-media or gaming-related phenotype relevant to the review, (ii) did not address genetic, endocrine or related biological mechanisms, (iii) were animal or in vitro studies without direct relevance to the human evidence reviewed, (iv) were editorials, commentaries, conference abstracts or other non-original publications, or (v) did not provide sufficient information regarding the investigated phenotype or biological variable.

Because this article is a narrative review rather than a systematic review or meta-analysis, study selection was guided by the relevance of the available evidence to the biological pathways examined in the review. Studies were not selected solely on the basis of statistical significance. Particular attention was given to studies that investigated specific candidate genes or biological pathways discussed in the mechanistic sections of the review, provided clearly defined phenotypes and contributed relevant human evidence regarding genetic susceptibility or endocrine/neurobiological mechanisms.

## 3. Results

The literature search retrieved relevant evidence addressing genetic, epigenetic, endocrine and neurobiological factors potentially associated with problematic digital-media use and gaming-related behaviors. The included evidence comprised predominantly observational human studies, with substantial heterogeneity in study populations, phenotypic definitions, genetic variants, and biological pathways investigated. The principal findings are synthesized according to the mechanistic domains addressed in the review.

The studies presented in Table 1 were selected to provide representative human evidence across the principal biological pathways examined in this review, including dopaminergic signaling (*DRD2/ANKK1*, *COMT*, and *DAT1*), serotonergic signaling, stress-related HPA-axis pathways (*CRHR1* and *FKBP5*) and other candidate pathways, such as *NTRK3*.

### 3.1. Dopamine and Reward System Genes

#### 3.1.1. Dopamine and Reward-Related Processes

One of the most studied biological systems in addiction is the dopaminergic reward pathway. Dopamine is a neurotransmitter, which is involved in motivation, pleasure, reward prediction, motivational salience, cue-triggered wanting and reinforcement learning. The mesolimbic system, also referred to as the brain’s reward system, which includes dopaminergic projections from the ventral tegmental area (VTA) to the nucleus accumbens and other limbic and cortical regions, contributes to the attribution of motivational significance to salient stimuli and to the reinforcement of behaviors [5,6].

Digital platforms and online games can provide frequent, salient, and sometimes unpredictable rewards, including notifications, likes, comments, social feedback, in-game rewards, and other variable digital reinforcers. These features may engage dopaminergic processes involved in reward prediction, motivational salience, cue-triggered wanting and reinforcement learning, potentially strengthening repeated engagement with digital activities. With repeated exposure, digital cues may acquire motivational significance and contribute to increasingly habitual patterns of checking, scrolling, or gaming, particularly in vulnerable individuals [5,6]. In addition, negative affective states such as stress, boredom, loneliness, and anxiety may promote continued engagement through negative reinforcement, further maintaining problematic patterns of use [11].

A relevant comparative perspective is provided by research on food addiction and highly palatable foods. Experimental and clinical research has identified several neurobiological and behavioral similarities between excessive consumption of highly palatable foods and addictive processes, including involvement of dopamine-related reward mechanisms [12]. However, these similarities should not be interpreted as evidence that food-related behaviors and digital-media use are clinically equivalent. Rather, the food-addiction literature provides a useful model for understanding how repeated exposure to highly salient rewards and reward-associated cues may contribute to persistent reward-seeking behavior.

Adolescence may represent a period of increased vulnerability to problematic digital-media use and gaming-related problems, because reward-related and motivational systems undergo substantial developmental changes while cognitive-control processes continue to mature. Individual differences in genetic and environmental factors may further contribute to susceptibility.

#### 3.1.2. *DRD2*

The dopamine D2 receptor gene (*DRD2*) has been investigated in relation to addictive behaviors, including gaming and compulsive internet use. Dopaminergic adaptations have been proposed as one possible mechanism underlying persistent engagement with highly reinforcing activities. In this context, prolonged exposure to rewarding stimuli has been hypothesized to alter D2 receptor-related signaling and responsiveness. Reduced sensitivity within the D2 receptor system (downregulation) has been proposed as one potential consequence of repeated stimulation of reward pathways and may contribute to diminished responsiveness to naturally rewarding experiences and increased motivation to pursue more salient or immediately reinforcing stimuli [13]. Altered dopaminergic signaling may also influence motivational processes, decision-making, and the development of habitual behavioral patterns associated with excessive Internet use. Among the variants most frequently associated with problematic digital-media use is the *DRD2/ANKK1 Taq1A* polymorphism (rs1800497). This SNP involves a C > T substitution and is located about 10 kb downstream of *DRD2*, within the adjacent ankyrin repeat and kinase domain containing 1 (*ANKK1*) gene. Subsequent molecular characterization established that the variant is located in exon 8 of *ANKK1* and results in a Glu713-na-Lys substitution in the putative ANKK1 protein [14]. Although rs1800497 is located within *ANKK1* gene, in close proximity to *DRD2*, it has historically been referred to as the “*DRD2 Taq1A*” polymorphism because it was originally identified as a polymorphism associated with the *DRD2* locus. Subsequent characterization demonstrated that the variant is located within *ANKK1* [15]. The A1 allele has been associated in some studies with reduced *DRD2* gene expression [14]. Associations between this allele and problematic digital-media behaviors have also been investigated. For example, studies have reported differences in A1 allele frequency among male adolescents with Internet gaming disorder and have examined its relationship with excessive reward-seeking tendencies [13]. Beyond problematic digital-media use, rs1800497 has been investigated across a range of behavioral and psychiatric phenotypes, including substance abuse, alcohol dependence, eating disorders, smoking cessation [14] and cognitive flexibility in individuals exhibiting pathological gambling-related behaviors [13].

#### 3.1.3. *COMT*

The COMT (catechol-O-methyltransferase) enzyme is involved in the metabolism of catechol compounds, such as dopamine, norepinephrine, and catechol-estrogens [16]. The COMT genotype on chromosome 22q11.2 is a G-to-A missense single-nucleotide polymorphism encoding either valine (Val) or methionine (Met) at codon 158 and is named the *COMT Val158met* polymorphism (rs4680) [16,17]. This functional variant influences COMT enzymatic activity and may consequently affect catecholamine availability, particularly dopamine regulation in the prefrontal cortex. Its potential contribution to addictive and reward-related behaviors has therefore been investigated [16]. The *Val158Met* polymorphism results in three genotypic groups with different levels of COMT enzymatic activity:

1. Val/Val (High Activity): Individuals homozygous for the Val allele generally exhibit higher COMT activity and consequently greater dopamine metabolism. This may be associated with relatively lower prefrontal dopamine availability under certain conditions. The Val/Val genotype has been investigated in relation to novelty-seeking, dependence and impulsive behaviors, such as gambling and risky decision-making.
2. Met/Met (Low Activity): Individuals homozygous for the Met allele generally exhibit lower COMT activity and reduced dopamine metabolism, which may result in relatively greater dopamine availability in the prefrontal cortex. Consequently, the Met allele has been associated with aspects of cognitive and executive functioning.
3. Val/Met (Intermediate Activity): Heterozygous individuals generally exhibit intermediate COMT enzymatic activity and may therefore show an intermediate pattern of dopamine metabolism [17].

A South Korean study involving 154 male adolescents (79 with excessive Internet video game use and 75 age- and sex-matched controls) investigated the combined effects of rs1800497 (*DRD2* gene, *Taq1A1* allele) and rs4680 (methionine variant of *COMT* gene) in relation to Internet gaming behavior. The authors reported an association between the combination of these variants and problematic Internet gaming, proposing a relationship with dopaminergic characteristics [18]. Other studies have also investigated these variants in relation to alcohol use, gambling-related behaviors and ADHD [19].

#### 3.1.4. *DAT1*

The dopamine transporter (DAT) is a key player in dopamine neurotransmission and is a protein located at nerve terminals which modulates the dynamics and levels of released dopamine (by recycling extracellular dopamine back into the presynaptic terminal, thus terminating its action). Dysregulated dopamine activity may stem from altered release or reuptake. Therefore, proper regulation of *DAT1* gene expression is critical to maintain homeostasis in the dopaminergic systems. The human *DAT1* gene, coding for DAT protein, has a polymorphic 40-base pair (bp) variable number of tandem repeats (VNTR) in the 3′-untranslated region (3′-UTR) of the dopamine transporter gene (*SLC6A3*, on chromosome 5p15.3). Even if the 40 bp VNTR element can be repeated 3–11 times, the greatest frequency polymorphisms are 9- or 10-repeat alleles. Both in vitro and in vivo studies demonstrated that gene expression was greater for the 10-repeat allele than for the 9-repeat allele, showing that this polymorphism can affect dopamine dynamics through protein expression. This polymorphism was previously reported to be associated with impulsivity and drug dependence/addiction. In addition, studies showed a correlation between the *DAT1* gene, in terms of protein functioning and promoter methylation, with excessive and compulsive use of Internet-based resources, while differences in *DAT1* genotype and promoter methylation have been examined in association with reward and sensation seeking, aggression and affiliative behaviors, and ADHD [20]. In a cross-sectional study of 79 Italian adolescents, interactions were observed between *DAT1* methylation levels, 5′-UTR/3′-UTR genotypes, impulsivity, and attachment-driven relationship quality, suggesting a possible association between epigenetic regulation of *DAT1* and individual vulnerability to Internet addiction [20].

#### 3.1.5. *DRD4*

The dopamine D4 receptor gene (*DRD4*), located on chromosome 11p, is characterized by substantial genetic variability. One of its most extensively studied variants is a 48 bp Variable Number Tandem Repeat (VNTR) located in exon 3, with alleles ranging from 2 to 11 repeats. The 2-repeat (2R), 4-repeat (4R), and 7-repeat (7R) variants are among the most frequently observed alleles. The polymorphic region encodes part of the third intracellular loop of the D4 receptor and can influence receptor signaling properties. Allele frequencies vary considerably across populations, with the 4R allele generally being the most prevalent, whereas the distribution of the 2R and 7R alleles differs substantially between populations. Functional studies have suggested differences in dopamine-related receptor activity across *DRD4* repeat variants. In particular, the 7R variant has been reported to exhibit lower dopamine-mediated signaling than the 2R and 4R variants in experimental systems. The 7R variant has been examined in connection with a range of psychiatric conditions, including ADHD, dependences, pathological gambling, alcoholism, drug dependence and bulimia nervosa. Variation in the length of the *DRD4* VNTR has also been associated with behavioral and personality traits such as externalizing behavior, novelty seeking, impulsivity, anger, irritability, thrill seeking and aggressive or delinquent behavior [21]. However, previous studies have reported an association involving the *DRD4* 4R genotype, attentiveness, and Internet addiction-related behavior [22].

### 3.2. Cortisol and Hypothalamic–Pituitary–Adrenal (HPA) Axis Genes

#### 3.2.1. Overview of the HPA Axis

Hypothalamic–pituitary–adrenal (HPA) axis is an intertwining of the central nervous system and endocrine system and is a major neuroendocrine regulator of the stress response, integrating neuroendocrine, immune, metabolic, and behavioral adaptations to internal and external stressors, such as chronic psychological stress, nutritional imbalances, disrupted circadian rhythms and environmental exposures [23]. Stress stimulates the release of corticotropin-releasing hormone (CRH) from the hypothalamus, then CRH stimulates secretion of adrenocorticotropic hormone (ACTH), and ACTH stimulation produces cortisol. Cortisol triggers negative feedback in the HPA axis [24]. Alterations in HPA-axis activity have been reported in association with addiction-like behaviors, including excessive social media use. Chronic activation of the HPA axis causes sustained cortisol to increase and altered stress responses. This chronic stress reaction may contribute to maladaptive behaviors and may increase vulnerability to addictive habits. Excessive screen time, particularly social media use, has been associated with alterations in HPA axis activity, which may resemble stress-related dysregulation observed in substance use disorders. The prolonged activation of the HPA axis in these contexts has been associated with changes in stress responsivity and can interact with emotional regulation, cognitive processes, reward-related mechanisms, and impulsivity, potentially contributing to the persistence of maladaptive behaviors. Moreover, chronic cortisol elevation may adversely affect immune function and may contribute to fatigue and sleep disturbances [10,24]. Genetic variations affecting this system could contribute to individual differences in stress responsivity and vulnerability to addictive behaviors during periods of chronic stress or emotional instability.

#### 3.2.2. *CRHR1*

The *CRHR1* gene encodes the G-protein coupled type I CRH receptor (CRHR1), a protein that is essential for the activation of signal transduction pathways that activate mesolimbic and HPA axis responses to many types of stress [25]. The CRHR1 receptor is crucial for establishing the initial HPA axis response to stressful events [24]. Several specific SNPs within the *CRHR1* gene have been linked to distinct clinical phenotypes and behavioral outcomes. *CRHR1* polymorphisms, such as rs110402, rs242924 and rs7209436, have been associated with major depression and suicidal behavior in interaction with childhood maltreatment or adverse experiences. An interaction between *CRHR1* and stressful life events has also been shown to influence alcohol use (rs242938, rs1876831) [25,26]. In specific populations, haplotype combinations (like the GGA haplotype involving SNPs such as rs4458044 and rs242924) have been associated with increased susceptibility to aggressive behaviors [27]. In a South Korean study involving 230 male adolescents, the *CRHR1* rs28364027 polymorphism was associated with Internet Gaming Disorder. Carriers of the AA genotype showed 2.69-fold higher odds of IGD compared with G-allele carriers (OR = 2.69, 95% CI: 1.17–6.15) [24].

#### 3.2.3. *FKBP5*

Whereas CRHR1 helps establish the initial HPA axis response to a stressful episode, the FK506 binding protein 5 (FKBP5) assists in terminating the stress response [25]. FKBP5 is a co-chaperone of the glucocorticoid receptor (GR) heterocomplex, part of the negative feedback loop regulating GR activity. When cortisol binds to the GR, FKBP5 is replaced by a positive regulator of GR signaling, FKBP4, and the complex is shuttled into the nucleus for transcriptional regulatory activity. Overexpression of FKBP5 reduces nuclear translocation of the GR complex by sequestering it in the cytosol. Interestingly, glucocorticoid exposure increases expression of FKBP5, dampening glucocorticoid negative feedback and allowing cortisol to participate in its own regulation. Alterations in the expression or function of FKBP5 could increase cortisol burden and contribute to the allostatic shift in cortisol regulation that occurs with repeated exposure to stress. Indeed, multiple polymorphisms in the *FKBP5* gene, such as rs3800373, rs4713916 and rs1360780 (T-allele), have been associated with mood disorder and response to antidepressant treatment, while variation in *FKBP5* has also been shown to interact with child abuse or trauma to predict adult depression, suicidality and bipolar disorder (rs4713902) [25,28]. In a longitudinal study of 1592 Chinese early adolescents, childhood maltreatment predicted increased problematic Internet game use over time, with internalizing symptoms acting as a mediator and *FKBP5* genetic variation (cumulative genetic score (CGS) based on four *FKBP5* SNPs: rs4713916, rs1360780, rs3800373, and rs9296158), moderating individual susceptibility to this association (interaction effects *p* < 0.05) [29].

**Table 1:** Representative original human studies addressing genetic and endocrine pathways in problematic digital-media use and gaming-related behaviors.

Reference	Study Design	Focus	Main Results
Rył et al. (2024) [13]	Cross-sectional genetic association study (Poland)	*ANKK1*, *DRD2*, and *NTRK3* polymorphisms	N = 396 healthy young men. *ANKK1* rs1800497 was significantly associated with risky/problematic Internet use, with A1 allele carriers showing higher PIU scores (*p* < 0.05). No significant associations were found for *DRD2* rs1076560 or *NTRK3* rs2229910 (*p* > 0.05).
Yen et al. (2022) [16]	Case–control genetic association study (Taiwan)	*COMT* polymorphism and Internet Gaming Disorder	N = 207 participants (69 with Internet Gaming Disorder [IGD] and 138 healthy controls). Individuals with the *COMT* Val/Val genotype had significantly higher odds of Internet Gaming Disorder than Met allele carriers (OR = 2.09, 95% CI: 1.15–3.80). The association between the *COMT* rs4680 polymorphism and IGD was mediated by impulsivity and fun-seeking.
Renshaw et al. (2007) [18]	Case–control genetic association study (South Korea)	*DRD2 Taq1A1* and *COMT Val158Met* polymorphisms in relation to reward dependence.	N = 154 male adolescents (79 with excessive Internet video game play [EIGP] and 75 age-matched healthy controls). The *DRD2 Taq1A1* allele (*p* = 0.03) and the low-activity *COMT* allele (Met) (*p* = 0.04) were significantly more prevalent in adolescents with excessive Internet video game play than in controls.
De Nardi et al. (2020) [20]	Cross-sectional genetic study (Italy)	*DAT1* methylation and polymorphisms	N = 79 young adults. Higher Internet Addiction scores were associated with altered *DAT1* methylation patterns, particularly interactions between 5′-UTR and 3′-UTR *DAT1* genotypes (*p* < 0.05). *DAT1* methylation levels showed significant correlations with impulsivity measures and attachment-related relationship quality (*p* < 0.05).
Cerniglia et al. (2020) [22]	Cross-sectional genetic association study (Italy)	Genetic polymorphisms and family functioning	N = 104 young adults. Results of mediation analyses showed a mediation role played by depressive symptoms (B = 0.99; 95% CI 0.22 to 1.97) and avoidant personality problems (B = 1.09; 95% CI 0.32 to 2.05) of young adults on the relationship between the Family Assessment Device (FAD), Adult Self-Report (AR), and Internet addiction. Finally, this relationship was moderated by the genotype of the 5-HTTLPR (*p* < 0.001), *DAT1* (*p* < 0.001), and *MAO-A* (*p* < 0.001) genes in young adults.
Park et al. (2018) [24]	Cross-sectional Genetic association study (South Korea)	*CRHR1* polymorphism	N = 230 Korean male adolescents (Internet Gaming Addiction group and control group). The *CRHR1* rs28364027 polymorphism was significantly associated with Internet Gaming Addiction. The AA genotype was more frequent in the IGA group and was associated with increased risk of IGA compared with G-allele carriers (OR = 2.69; 95% CI: 1.17–6.15). The AA genotype (*p* = 0.016) and A allele (*p* = 0.021) showed significant associations with IGA susceptibility.
Gong X, Zhou J. (2025) [29]	Longitudinal genetic association study (China)	Childhood maltreatment, internalizing symptoms, problematic Internet game use (PIGU), and *FKBP5*-related cumulative genetic scores (CGS)	N = 1592 Chinese early adolescents. Childhood maltreatment was positively associated with subsequent problematic Internet game use. Internalizing symptoms significantly mediated the relationship between childhood maltreatment and PIGU. The *FKBP5* cumulative genetic score significantly moderated the association between emotional abuse and internalizing symptoms: high genetic risk group b = 0.13, SE = 0.03, *p* < 0.01.

ANKK1: ankyrin repeat and kinase domain containing 1; DRD2: dopamine receptor D2; NTRK3: neurotrophic receptor tyrosine kinase 3; PIU: problematic Internet use; COMT: catechol-O-methyltransferase; OR: odds ratio; CI = confidence interval; IGD: Internet gaming disorder; EIGP: excessive Internet video game play; DAT1: dopamine transporter 1; UTR: untranslated region; MAO-A: monoamine oxidase A; CRHR1: corticotropin-releasing hormone receptor 1; AA: homozygous for the A allele; IGA: Internet gaming addiction; PIGU: problematic Internet game use; FKBP5: FKBP prolyl isomerase 5 (or FK506 binding protein 5); SE: standard error.

### 3.3. Other Hormonal and Genetic Factors

#### 3.3.1. Serotonin and *SLC6A4*

Serotonin (5-HT) plays a major role in feelings of well-being, happiness, anxiety, stress susceptibility, and depression [22]. The serotonin neurotransmitter pathways ensure central brain control of the rhythm of sleep/wake periods and the immune response of the whole organism [30]. Studies of individuals with Internet Gaming Disorder (IGD) have reported decreased striatal dopamine availability and reduced serotonin availability [30]. After its vesicular release, the level of serotonin is maintained by the serotonin transporter (5-HTT) that actively clears it from the synapse, returning it to the presynaptic neuron where it can be degraded or released later. The serotonin transporter is encoded by a single gene [the solute carrier family 6, member 4 (*SLC6A4*) gene] located on chromosome 17q11.1-17q12 and spans 31 kb. It consists of 14 exons and belongs to a large gene family of neurotransmitter transporters, which have a sodium- and chloride-dependent transport activity, and the transporter contains 12 transmembrane domains. The transcription of this gene is modulated by two primary polymorphisms in its upstream regulatory region. The first polymorphism is a 44 bp insertion deletion located in the promoter region (5-HTTLPR), creating the long and the short variants of the gene. The long variant (L) indicates the presence of an insertion, resulting in a 528 bp allele having 20–23 bp long, GC-rich 16 repeat elements, whereas the absence of this insertion yields a short variant (S) of 484 bp allele having 14 repeat elements. The L allele confers a threefold increase in transcriptional efficacy and activity than the S allele. Serotonin transporter-linked polymorphic region (5-HTTLPR) is one of the most extensively studied polymorphisms in psychiatric behavioral genetics [31]. Studies have reported association between Internet Addiction and the S/S genotype (SS-5HTTLPR, rs25531). As was supported by numerous studies, the aforementioned genetic variants were also associated with a predisposition to depression, the most prevalent comorbid disorder in Internet-addictive subjects [19].

#### 3.3.2. Other Genetic Factors

1. *CHRNA4* (nicotinic acetylcholine receptor subunit alpha 4 gene—rs1044396): The CC genotype has been investigated in relation to nicotine addiction and attention disturbances [19]. In a case–control study of Korean male adults, the rs1044396 polymorphism was associated with Internet Gaming Disorder (IGD). Specifically, the T allele was less frequent among individuals with IGD, while carriers of the CT and TT genotypes showed lower addiction severity. These findings suggest a possible association between the T allele and lower severity of problematic gaming [32].
2. *NTRK3* (neurotrophic tyrosine kinase receptor type 3 gene—rs2229910): This polymorphism has been associated with anxiety and depressive disorders, obsessive-compulsive disorders, and psychologically determined nutrition diseases [13]. According to a study, a significant difference in the minor allele frequency was observed between individuals with Internet Addiction and controls (*p* = 0.019). The *NTRK3* polymorphism was associated with lower odds of problematic Internet use (PIU), suggesting a possible association with reduced susceptibility (OR = 0.154) [33].
3. GDNF (glial cell-derived neurotrophic factor): GDNF is a neurotrophic factor that has an important role in the maintenance of dopaminergic neurons in several brain regions, as well as in the development, survival, and maintenance of dopaminergic neurons in the midbrain. In individuals with Internet Gaming Disorder, plasma GDNF levels have been reported to be lower than those observed in controls and inversely associated with severity of internet addiction and motivation-related cognitive processes [34]. The polymorphisms rs1549250 and rs2973033 of *GDNF* gene have been investigated in relation to multiple addictive behaviors, including lifetime drug use. These findings suggest that *GDNF*-related genetic variation may contribute to individual differences in susceptibility to addictive behaviors [35].
4. MAO-A (monoamine oxidase A): The *MAO-A* gene has a 30-base pair repeat in the promoter region that affects transcriptional efficiency. The high-activity variant (*MAOA-H*, 3.5 or 4 repeats) has been associated in some studies with impulsive personality traits and tobacco and cannabis use. However, findings across studies have been inconsistent, with the low-activity variant (*MAOA-L*, 2, 3, or 5 repeats) also being associated with disordered gambling [22].

### 3.4. Sleep and Circadian Pathways

Nighttime exposure to light from electronic devices has been associated with disruption of melatonin-dependent circadian rhythms, contributing to sleep disturbances. Melanopsin, a photosensitive protein in the retina, has long been shown to drive melatonin levels to entrain our circadian rhythm [36]. Exposure to blue light from screens thereby influences melatonin secretion through the action of melanopsin [36], although the magnitude of this effect depends on factors such as light intensity, spectral composition, exposure duration and timing relative to the individual’s circadian phase [37]. In addition, substantial interindividual differences exist in sensitivity to evening light and in the magnitude of melatonin suppression [38]. Plasma melatonin levels are typically low during the day and elevated at night due to rapid metabolism, as part of the normal circadian pattern [36].

In cases of problematic digital-media use, difficulty disengaging from electronic devices at night may prolong exposure to display light, which can alter melatonin secretion and circadian timing and potentially contribute to sleep difficulties or reduced sleep quality [36].

## 4. Discussion

The findings of the present review support the growing body of evidence suggesting that problematic digital-media use and gaming disorder are multifactorial conditions shaped by the complex interaction of biological, psychological, and environmental determinants. Rather than representing isolated behavioral problems, these conditions may share some neurobiological and psychosocial characteristics with substance-related addictions, reinforcing their recognition as significant public health concerns [10].

One of the principal findings emerging from the reviewed literature is the potential contribution of genetic susceptibility to problematic Internet and gaming use. Polymorphisms in dopamine-related genes, including *DRD2*, *COMT*, and *DAT1*, have been investigated in relation to reward processing, impulsivity, and executive functioning, potentially contributing to individual differences in vulnerability to problematic digital-media use and gaming-related behaviors. Nevertheless, genetic predisposition alone is insufficient to explain the onset of problematic digital-media use, highlighting the importance of environmental influences, family dynamics, and individual psychological characteristics in determining clinical outcomes [13,16,19,20].

The reviewed evidence also emphasizes the potential involvement of neurobiological mechanisms in problematic digital-media use. Alterations in dopaminergic signaling and reward-related processing have been reported in individuals with problematic Internet and gaming use. Rather than being attributed solely to persistent dopaminergic overstimulation, these findings can be considered in the context of contemporary models of addiction that incorporate reward prediction, cue-triggered motivational salience, reinforcement learning, habit formation, and impaired control [5,6,13].

In addition to alterations in dopaminergic signaling, hormonal and neuroendocrine processes may represent another important biological pathway associated with problematic digital-media use. Alterations in hypothalamic–pituitary–adrenal (HPA) axis activity and cortisol responses have been reported in association with psychological stress and problematic patterns of digital-media use [10,23]. Similarly, nighttime exposure to light from electronic devices may alter melatonin secretion and circadian timing, potentially contributing to sleep disturbances and reduced sleep quality [36]. Given the established relationships among sleep, emotional regulation, and cognitive functioning, these physiological processes may interact with psychological and behavioral factors and potentially contribute to the persistence of problematic digital-media use.

Another important observation is the strong bidirectional association between problematic digital-media use and psychiatric disorders, particularly depression, anxiety disorders, and attention-deficit/hyperactivity disorder (ADHD). Current evidence suggests that these conditions may both represent vulnerability factors for problematic Internet and gaming use and be associated with psychological difficulties that emerge or worsen in the context of excessive digital engagement. This reciprocal relationship highlights the importance of comprehensive clinical assessment, as the identification and treatment of coexisting psychiatric symptoms may improve the effectiveness of interventions targeting problematic Internet and gaming use [10].

Importantly, literature consistently identifies several protective factors that may be associated with lower levels of problematic digital-media use. Supportive parenting practices, effective parental monitoring, positive interpersonal relationships, and regular physical activity appear to enhance psychological resilience and self-regulation and may therefore represent potential protective factors against problematic Internet and gaming use. These findings emphasize that preventive strategies should extend beyond restricting screen time and instead promote healthy family environments, social connectedness, and adaptive coping mechanisms [10].

Despite the increasing volume of research, several limitations remain within the existing literature. Most of the identified candidate-gene studies were conducted in relatively small and restricted populations, with several studies focusing predominantly on Asian male adolescents, which may limit the generalizability of their findings. In addition, many studies used cross-sectional designs, heterogeneous phenotypes and assessment instruments, and relatively limited adjustment for potential confounders and multiple comparisons. Independent replication of reported associations remains limited, and the available candidate-gene findings have generally not reached genome-wide significance. Furthermore, functional interpretations of individual polymorphisms are often based on indirect evidence and may not fully reflect their biological effects in the context of problematic digital-media use. Accordingly, these genetic findings should be interpreted cautiously as preliminary and hypothesis-generating signals rather than as established causal determinants or clinically actionable biomarkers.

Future studies should therefore employ adequately powered sample sizes determined through formal power calculations and include well-characterized and diverse clinical and control populations using standardized diagnostic criteria and validated assessment instruments. Multicenter and longitudinal designs would be particularly valuable for clarifying temporal and potentially causal relationships. Genetic studies should incorporate appropriate adjustment for potential confounders and multiple testing, preferably using genome-wide approaches, and findings should be replicated in independent cohorts. Where feasible, preregistration and transparent reporting of study protocols and analytical plans should be encouraged. Objective behavioral measures, alongside genetic, endocrine, neurobiological, and clinical assessments, could further strengthen the characterization of relevant phenotypes. Such approaches will be essential to determine whether the biological associations identified to date are reproducible and clinically meaningful and whether they may ultimately contribute to risk prediction or personalized interventions.

Overall, the available evidence suggests that problematic digital-media use and gaming disorder should be understood as complex phenomena, influenced by interacting biological, psychological, and environmental factors, and may benefit from multidisciplinary approaches to prevention, early identification, and clinical management. Integrating biological, psychological and environmental perspectives may contribute to the development of more effective clinical interventions and evidence-based public health policies aimed at reducing the growing burden of problematic digital-media use among adolescents.

## 5. Conclusions

Internet, smartphone, and gaming-related problematic behaviors are complex phenomena influenced by interacting psychological, environmental, genetic, and endocrine factors. Current evidence suggests that individual differences in dopaminergic regulation, stress-response systems, serotonin signaling, and hormonal pathways may be associated with vulnerability to problematic digital-media use. Although digital technology offers substantial benefits, excessive and uncontrolled use may be associated with changes in neurobiological processes involved in reward, stress, sleep, and emotional regulation. Adolescents may represent a particularly vulnerable population, although the contribution of specific genetic and endocrine factors to individual susceptibility remains incompletely understood. Future research integrating neuroscience, genetics, endocrinology, and psychology will be essential to clarify the biological mechanisms and their potential clinical relevance. Large, adequately powered and preregistered longitudinal studies using harmonized phenotypes, objective behavioral measures, genome-wide approaches, and independent replication are needed before genetic or endocrine markers can be considered for risk prediction or personalized interventions. A deeper understanding of the biological pathways associated with problematic digital-media use may ultimately contribute to more effective prevention and clinical management strategies and to healthier relationships with digital technology.

## Data Availability

No new data were created or analyzed in this study. Data sharing is not applicable to this article.
